# International Clinical Trials Day 2014—ancient and modern needs

**DOI:** 10.3332/ecancer.2014.ed35

**Published:** 2014-05-20

**Authors:** Francesco Perrone

**Affiliations:** Clinical Trials Unit, National Cancer Institute of Naples, Italy

Today is International Clinical Trials' Day, which is always celebrated around the 20th of May, the day during the Spring of 1747 when James Lind began a clinical trial that produced useful information to understand, treat and prevent scurvy. The reason why many of us still dedicate a celebration to James Lind goes well beyond the intrinsic value of his trial, which actually had many flaws and would not be suitable for registering a new drug in modern times. We refer to the story of his 12 scorbutic sailors, divided into 6 couples each treated in a different way, as the inauguration of clinical trials methodology, something we still use and need today.

I’m an oncologist and please don’t blame me if I’ll be biased by the field where I work.

Underpinning my considerations lies a strong similarity: scurvy was a plague in James Lind’s microenvironment during the 18th century, cancer is a plague in the microenvironment of Western rich countries at beginning of the 21^st^, thanks to the successful fight against infectious disease [1]. But the needs of modern medicine are becoming more and more complicated. Among the modern challenges of cancer treatment, two seem to me to be extremely important and both include clinical trials as the core methodology: innovation and affordability - the sunny and the shady sides of the street.

Hopefully, a high number of new drugs will be produced in the next few years, more and more precisely directed towards targets that are crucial in some way for the survival of cancer cells. This revolution will produce undeniable benefits for the patient, but is already challenging scientists and regulatory bodies because of methodological problems based on the development of appropriate biomarkers, validation of biomarkers and surrogate end-points, and the fact that molecular characterization will transform frequent types of cancer in a number of rare diseases [2], creating difficulties for performing clinical trials of the right size [3]. Methodological – e.g. adaptive clinical trial designs – and regulatory – e.g. conditioned or temporary approval based on early clinical trials while looking for more definitive ones - reactions are needed to let innovation enter into clinical practice with solid evidence about safety and efficacy. More and more very early clinical trials are changing their faces; phase 1 studies often allow solid evidence of activity and particularly in the case of rare tumours (or frequent tumours with rare mutations) it is no longer crazy to think about registration based on the results of phase 2 or even of phase 1 trials [4].

Unfortunately, the price of drugs has already dramatically increased during the last few years, even with drugs that produced only small incremental benefits [5]. Once again, clinical trials are the cornerstone, both for raising the bar of efficacy during the development of new drugs [6] and to provide to regulatory and governmental bodies evidence on relative efficacy that may inform economic strategies. Pragmatic clinical trials, promoted and performed independently by academic investigators, often represent the most ethical way to distinguish between true and fatuous progress, and we should do our best to make the former available to all the patients who can benefit. In this field, phase 3 randomised trials and phase 4 studies still remain the cornerstone of methodology to be applied. Large sample sizes are required to properly assess relative efficacy of available treatments and generalizability and rapid impact of such trials into clinical practice will increase as more they are conducted with simplified procedures. Academic Institutions, cooperative groups, and governmental bodies should play the role of promoter in many of these studies that do not necessarily meet the priorities of pharmaceutical industries. Great attention should also be paid to patient perception of clinical benefit, as well as to patient perception of toxicity [7].

Alas, it is no longer a story of oranges and lemons! But we should continue to be inspired by James Lind’s story to tackle modern clinical trials challenges to the best of our enthusiasm within the realms of possibility.
